# Network dynamics underlying alterations in apparent object size

**DOI:** 10.1093/braincomms/fcae006

**Published:** 2024-01-09

**Authors:** Lihong Chen, Baoyu Wu, Haoyang Yu, Irene Sperandio

**Affiliations:** Research Center of Brain and Cognitive Neuroscience, Liaoning Normal University, Dalian 116029, China; Key Laboratory of Brain and Cognitive Neuroscience, Dalian 116029, Liaoning Province, China; Research Center of Brain and Cognitive Neuroscience, Liaoning Normal University, Dalian 116029, China; Key Laboratory of Brain and Cognitive Neuroscience, Dalian 116029, Liaoning Province, China; Department of Psychology and Behavioral Sciences, Zhejiang University, Hangzhou 310058, China; Research Center of Brain and Cognitive Neuroscience, Liaoning Normal University, Dalian 116029, China; Key Laboratory of Brain and Cognitive Neuroscience, Dalian 116029, Liaoning Province, China; Department of Psychology and Cognitive Science, University of Trento, Rovereto 38068, Italy

**Keywords:** Ebbinghaus illusion, feedback projection, fMRI, DCM

## Abstract

A target circle surrounded by small circles looks larger than an identical circle surrounded by large circles (termed as the Ebbinghaus illusion). While previous research has shown that both early and high-level visual regions are involved in the generation of the illusion, it remains unclear how these regions work together to modulate the illusion effect. Here, we used functional MRI and dynamic causal modelling to investigate the neural networks underlying the illusion in conditions where the focus of attention was manipulated via participants directing their attention to and maintain fixation on only one of the two illusory configurations at a time. Behavioural findings confirmed the presence of the illusion. Accordingly, functional MRI activity in the extrastriate cortex accounted for the illusory effects: apparently larger circles elicited greater activation than apparently smaller circles. Interestingly, this spread of activity for size overestimation was accompanied by a decrease in the inhibitory self-connection in the extrastriate region, and an increase in the feedback connectivity from the precuneus to the extrastriate region. These findings demonstrate that the representation of apparent object size relies on feedback projections from higher- to lower-level visual areas, highlighting the crucial role of top-down signals in conscious visual perception.

## Teaser

Chen *et al*. report that apparently larger objects elicit greater activation in extrastriate cortex and increase the feedback connectivity from the precuneus to the extrastriate region. The findings highlight the crucial role of top-down signals in conscious visual perception.

## Graphical Abstract

**Graphical Abstract:**
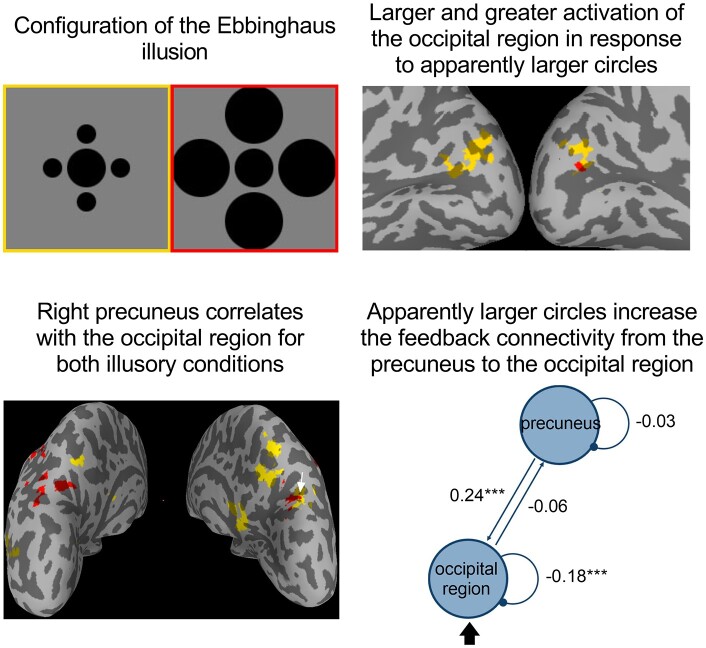

## Introduction

It is often said that ‘seeing is believing’, implying that what we see and what we believe we see are overlapping phenomena. However, visual illusions teach us an important lesson: subjective experience can depart from the physical sensation. One of these instances is the Ebbinghaus illusion, whereby the size of a central circle surrounded by an annulus of smaller circles is perceived as larger than a physically identical circle surrounded by an annulus of larger circles. Visual illusions, like the Ebbinghaus, are a key methodology in vision research as they provide a unique window into the mechanisms responsible for our subjective experience of the visual world.

Previous research suggests that both early and higher order visual areas might be involved in the processing of the Ebbinghaus illusion. For example, psychophysical evidence from interocular transfer studies indicates that the illusion relies at least in part on monocular neural populations.^1-3^ If one considers that monocular cells are mostly prevalent in subcortical visual areas, such as the lateral geniculate nucleus (LGN), and partially in the primary visual cortex (V1), it follows that the illusion should occur early in the visual pathway. Interestingly, Sperandio *et al*.^4^ demonstrated, using a variant of the Ebbinghaus illusion with flickering surrounding stimuli and a static central target able to induce an afterimage of the target only, that the afterimage of the target changed according to perceived size, as opposed to its veridical size, therefore excluding a retinal locus for the phenomenon. The involvement of V1 in representing the Ebbinghaus illusion has been further corroborated by brain imaging studies. It has been shown that inter-individual variability in illusion susceptibility could be predicted by variations in the local architecture of V1, such that susceptibility tended to decrease in individuals with greater central cortical magnification.^5,6^ One possible explanation for this finding is that in a large V1 the cortical distance between the target and its surrounding increases and, as a consequence, the influence of contextual information on size perception is reduced. More recently, Wang *et al*.^7^ used transcranial direct current stimulation and observed that when anodal stimulation was applied over the occipital cortex, the magnitude of the Ebbinghaus illusion was enhanced, presumably as a result of a weakening in the suppressive effect exerted by feedback projections from higher to lower visual areas on the surrounding context. Taken together, these findings suggest that V1 plays a critical role in perceived size by processing contextual information.

Beyond V1, there is evidence that higher order areas, such as the parietal cortex, play an important role too. In fact, Wu *et al*.^8^ reported that when activity of the right superior parietal lobule (SPL) was disrupted through repetitive transcranial magnetic stimulation, the effect of the Ebbinghaus illusion was temporarily increased. The authors also found, by applying dynamic causal modelling (DCM) on resting-state functional MRI (fMRI) data, that forward connections from right V1 to SPL could explain inter-individual differences in illusion susceptibility. Finally, a positive correlation has been reported between susceptibility to the Ebbinghaus illusion and different concentrations of GABA, an inhibitory neurotransmitter, in the right parietal cortex.^9^ Although the literature reviewed so far suggests that both early visual cortex and higher-level visual area are involved in the Ebbinghaus illusion, it remains unclear how the occipital and parietal areas interact to produce the illusion effect.

The current study aimed to investigate the neural networks underlying the Ebbinghaus illusion and characterize their effective connectivity by asking our participants to direct their attention to one of the two illusory configurations at a time. It has been shown that paying attention to the small or large surrounding circles can change the apparent size of the central target systematically: attending small contextual circles increased the perceived size of the central target, whereas attending large contextual circles decreased its perceived size.^10^ Therefore, we hypothesized that when both large and small contextual circles are presented together in an Ebbinghaus display, focusing attention on the large surrounding circles would induce size underestimation of the target, while focusing attention on the small surrounding circles would elicit size overestimation.

## Materials and methods

### Participants

A total of 36 healthy participants (14 males, mean age = 22.3 years, SD = 2.2) took part in the study: 16 were recruited for the behavioural experiment and another 20 for the fMRI experiment. Sample size was determined based on our previous studies of the Ebbinghaus illusion.^3,7^ All participants were right-handed, reported normal or corrected-to-normal vision and had no known neurological or psychiatric disorders. They were naïve to the purpose of the study, and gave written informed consent before taking part in the experiment in accordance with the Declaration of Helsinki. The study was approved by the institutional review board of Liaoning Normal University.

### Stimuli and procedure

**Figure 1:**
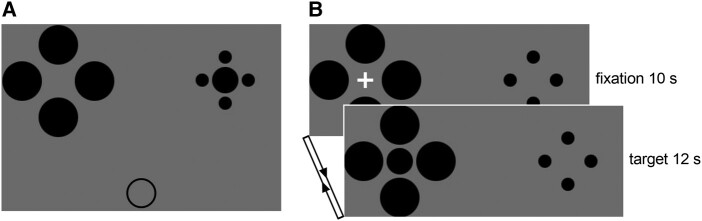
**Schematic representation of the experimental procedures.** (**A**) In the behavioural experiment, the stimuli consisted of four large and four small circles as well as a central target simultaneously presented in one display. A comparison circle was presented below the illusory configurations. (**B**) In the fMRI experiment, the illusory configurations and a white fixation cross were simultaneously presented for 10 s, then the fixation was replaced by the central target for 12 s.

Stimuli were generated and displayed using Matlab (Mathworks, Natick, MA) together with the Psychophysics Toolbox.^11,12^ For the behavioural experiment (Fig. 1A), four large (diameter = 1.7°) and four small (diameter = 0.6°) circles were respectively presented on the left and right sides of the screen simultaneously (6.8° from the screen centre), with their locations counterbalanced across trials. The central target circle (diameter = 1.1°) was presented either among the four large inducers or the four small inducers, and meanwhile a comparison circle was presented below the illusory configurations (8.3° from the screen centre). The diameter of the comparison circle was selected from one of 11 equally spaced values ranging from 0.8° to 1.4°. Participants were instructed to freely view the stimuli and adjust the size of the comparison circle to match that of the central target with no time limit. All stimuli were black and were presented on a grey background. Participants were positioned 57 cm away from the computer screen (1920×1080 at 60 Hz) with their head fixed on a chin rest. There were 44 trials in total, with 11 repetitions for each condition (layout of illusory configuration: large inducers on the left and small inducers on the right or vice versa; location of the central target: left or right).

The fMRI experiment consisted of four functional runs of 110 volumes each. Each run included 10 blocks, alternating 10 s fixation and 12 s target presentation (Fig. 1B). The contextual information (i.e. four small and four large inducers) was always presented on the screen, which allowed for direct comparison of overestimation and underestimation portion of the illusion effect while keeping the physical properties of the configurations constant. The position of the large and small inducers was counterbalanced between runs. Within each block, the fixation and the central target were always presented on the same location, which was counterbalanced across blocks. In order to avoid task- and motor-related brain signals, participants did not perform an explicit task during the fMRI session.^13^ They were simply required to maintain fixation on the cross and the central target. Visual angles of the stimuli were linearly scaled (factor = 1.5) to adjust sizes to the MRI setting.

### MRI data acquisition

In the MRI scanner, stimuli were back-projected via a video projector (1280×1024 at 60 Hz) onto a translucent screen placed inside the scanner bore. Participants viewed the stimuli through a mirror located above their eyes. Functional MRI data were collected using a 3 T scanner (MR-750, GE medical systems, Milwaukee, WI) with an eight-channel phase-array coil. BOLD signals were acquired using a T_2_*-weighted EPI sequence (echo time = 30 ms, repetition time = 2000ms, field of view = 224 mm, matrix = 64×64, flip angle = 90°, number of slices = 33, spatial resolution = 3.5×3.5×3.5 mm^3^). High-resolution 3D T_1_-weighted anatomic images (echo time = 3 ms, repetition time = 6.9 ms, field of view = 256 mm, matrix = 256×256, flip angle = 8°, number of slices = 176, spatial resolution = 1×1×1 mm^3^) were collected for each participant. To minimize head movements, straps and foam pads were used to fix the head comfortably during scanning. The first three scans were discarded to allow for magnetic field stabilization.

### MRI data processing and analysis

Image time-series were pre-processed and analysed using SPM12 (Statistical Parametric Mapping software, University College of London, London, UK; r7771). Images were slice-time corrected, spatially normalized into a standard stereotactic space (Montreal Institute on Neurology, MNI template) and smoothed using an isotropic 6 mm Gaussian kernel. Low frequency noise was removed via a high-pass filtering (cut-off 1/128 Hz), and time-series were corrected for serial autocorrelations using a first-order autoregressive AR(1) process. All coordinates were reported using the Montreal Neurological Institute (MNI) convention.

### Connectivity analysis

We applied an analysis of psychophysiological interaction (PPI) with a seed region in the occipital cortex to test for any changes in functional coupling between this seed region and each voxel in the whole brain as a function of the experimental conditions.^14^ Specifically, we tested for the rest of the brain showing higher functional coupling with the seed region when stimulated with illusory conditions versus fixation. The seed region was functionally localized based on a combined activation of illusory conditions relative to fixation including the occipital areas BA18 and BA17 (peak MNI: 11.5, −77, 14; cluster size = 49 voxels).

We extracted the first eigenvariate of the fMRI signal from the seed region. The experiment was expressed as a single regressor with illusory condition (i.e. the target presented among large or small inducers) minus control condition (i.e. fixation). PPI targeting whole brain regions were calculated with a general linear model that included the convolved PPI interaction term, the seed region time course, the experimental regressor convolved with HRF, the block regressor and a constant. After single-subject PPI analysis, we performed second level analysis with one-sample *t*-tests for each of the analysis separately, in which we used a threshold of *P* < 0.001, uncorrected, and a minimum cluster size threshold of *k* = 20 voxels, based on a standard grey mask.

### DCM analysis

Based on the results of PPI analysis, effective connectivities between the occipital region and the right precuneus were analysed using DCM in SPM12. The occipital region was activated by both illusory conditions, and the right precuneus showed significant connectivity with this occipital region for both illusory conditions. We summarized the BOLD signal in each participant using the first eigenvariate (principal component) of voxels within a sphere of 8 mm radius for the precuneus (MNI coordinates: 18.5, −80.5, 35), and using a cluster mask for the occipital region (MNI coordinates: 11.5, −77, 14; cluster size = 49 voxels). DCM models the hierarchical organization of the brain using self-connections within a region, as well as forward and backward connections between regions.^15^ The model space consisted of three models, which had the same basic architecture and differed with respect to the driving input region. The driving inputs included the illusory conditions. Effective connectivity analysis between the two regions was divided into (i) selecting the driving input region for the models and (ii) establishing a model by analysing the connectivities between the regions using parametric analysis. We used Bayesian Model Reduction to evaluate the evidence for these three models, and reported the posterior estimates under each model.

## Results

### Behavioural illusion effect

**Figure 2:**
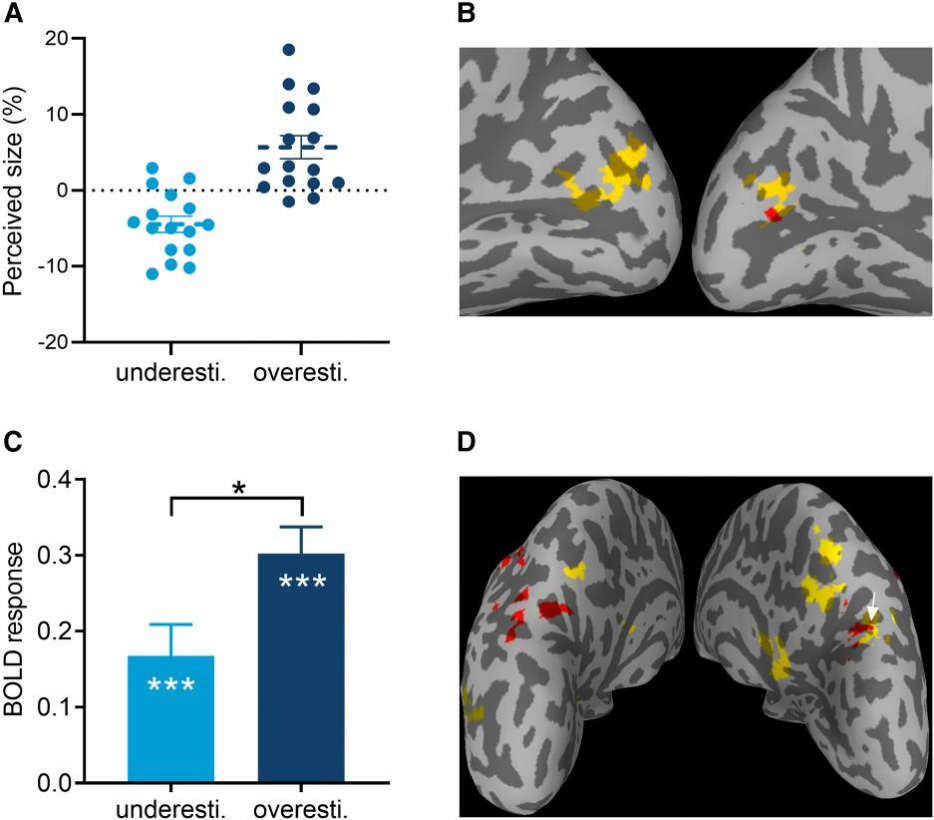
**Results of behavioural performance, group level GLM and PPI analysis.** (**A**) The perceived size of the central target (%) when presented among large inducers and small inducers (*n* = 16). (**B**) Brain activations in response to underestimation and overestimation conditions, and (**C**) BOLD response in the occipital region of interest (ROI) as a function of illusory conditions (*n* = 20, paired *t*-test). (**D**) Results of PPI analysis with the seed of the occipital ROI for the underestimation and overestimationconditions (*P* < 0.001, uncorrected, cluster size = 20 voxels). Underestimation is in red, and overestimation is in yellow. Error bars represent one standard error of the mean. Asterisks indicate a significance level of *P < 0.05, and ***P < 0.001. The white arrow indicates a common region (i.e. right precuneus) that showed correlation with the occipital ROI for both illusory conditions.

As predicted, participants underestimated the perceived size of the target when it was presented among large inducers (mean = −4.47%, SD = 4.25, *t*(15) = −4.21, *P* < 0.001, *d* = 1.05; Fig. 2A), and overestimated its perceived size when presented among small inducers (mean = 5.70%, SD = 6.10, *t*(15) = 3.73, *P* = 0.002, *d* = 0.93). Both the underestimation and the overestimation were unaffected by the location of the target (left versus right, *P* > 0.59). All the 16 participants showed a positive illusion effect (mean = 10.17%, SD = 4.33, *t*(15) = 9.40, *P* < 0.001, *d* = 2.35). The illusion effect was comparable when the large inducers were presented on the left side and the right side of the screen (*t*(15) = −0.19, *P* = 0.851, *d* = 0.05).

### Group level GLM

Relative to fixation, the overestimation condition (i.e. central target presented among small inducers) mainly activated occipital regions, including the lingual gyrus (peak MNI: 22, −63, −7), BA18 and BA17 (peak MNI: 11.5, −77, 14), whereas the underestimation condition (i.e. central target presented among large inducers) activated BA18 (peak MNI: −2.5, −87.5, 7; *P* < 0.05, voxel-wise FWE corrected; Fig. 2B).

### ROI results

We combined the occipital region (including BA17 and BA18, 49 voxels) activated by overestimation and underestimation conditions as a region of interest (ROI). Compared with fixation, both underestimation (*t*(19) = 4.08, *P* < 0.001, *d* = 0.92) and overestimation (*t*(19) = 8.72, *P* < 0.001, *d* = 1.95) conditions elicited greater activity in the occipital ROI. Notably, overestimation elicited larger activation than underestimation (*t*(19) = 2.55, *P* = 0.019, *d* = 0.57; Fig. 2C).

### PPI results

We performed PPI analysis with the seed of the occipital ROI (including BA17 and BA18; peak MNI: 11.5, −77, 14) using a grey mask and a threshold of *P* < 0.001, uncorrected, cluster size = 20 voxels. Activation positively correlated with size overestimation was observed in the left middle occipital gyrus, corpus callosum and right precuneus (Fig. 2D, Table 1). Activation positively correlated with size underestimation was observed in the left inferior putamen, left insula, left inferior frontal gyrus, right precuneus and bilateral inferior parietal lobule. The right precuneus region (parietal lobe, MNI coordinates: 18.5, −80.5, 35, 3 voxels) showed significant correlation with the occipital ROI for both illusory conditions.

**Table 1:** Results of PPI analysis with the seed of the occipital ROI

Condition	Region	Hemi.	Peak MNI coordinates			Cluster size
			*x*	*y*	*z*	
Overestimation	Middle occipital gyrus	L	−20	−84	10.5	23
	Corpus callosum	R	8	−38.5	3.5	27
	Precuneus	R	1	−70	52.5	89
Underestimation	Putamen	L	−30.5	0	−3.5	26
	Insula	L	−27	17.5	0	20
	Inferior parietal Lobule	L	−20	−73.5	42	63
	Precuneus	R	18.5	−77	35	20
	Inferior frontal gyrus	L	−48	3.5	35	36
	Inferior parietal lobule	R	39.5	−52.5	45.5	35

### DCM results

**Figure 3:**
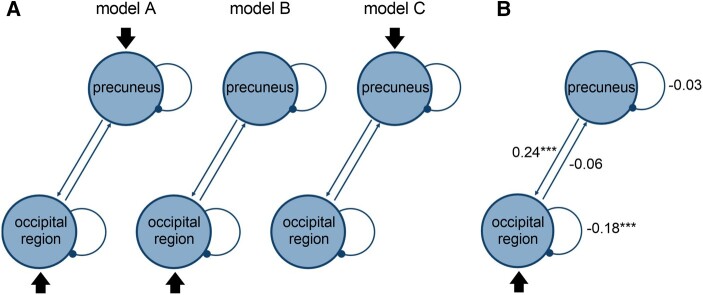
**Model space and DCM results.** (**A**) The model space consisted of three models, which had the same basic architecture and differed with respect to the driving input region. (**B**) The connection strength of the winning model was derived by comparing overestimation with underestimation condition (*n* = 20, paired *t*-test). The values indicate connectivity strength and asterisks indicate a significance level of ***P < 0.001.

Both animal and human studies have found bidirectional structural connections between the precuneus and the subcortical thalamus.^16,17^ Therefore, the driving input might enter either of the occipital ROI and the precuneus, or both regions. Three models, based on the connections between the occipital ROI and the right precuneus and the driving input region, were set up (Fig. 3A). The illusion effect (overestimation versus underestimation, without subtracting the corresponding fixation condition) was considered as a modulator of the connections between these sites and incorporated into the models.

As shown in Fig. 3, the evidence for Model B (posterior probability = 1) was higher than that for Models A and C. For the winning model (i.e. Model B), results of effective connectivity showed that the illusion effect was manifested by a significant decrease in inhibitory self-connection in the occipital region (paired *t*-test: *M* = −0.18, SD = 0.17, *t*(19) = −4.71, *P* < 0.001, *d* = 1.05) and a significant increase in feedback connection from the right precuneus to the occipital region (*M* = 0.24, SD = 0.20, *t*(19) = 5.35, *P* < 0.001, *d* = 1.20; Fig. 3B).

## Discussion

The current study investigated the neural mechanisms underlying the Ebbinghaus illusion, which was generated by using the classic ‘dual’ display: an annulus of big inducers and an annulus of small inducers were presented side by side. When participants focused their attention on the configuration with four large inducers, the perceived size of the central target was underestimated. In contrast, when they directed their attention to the configuration with four small inducers, the perceived size of the central target was overestimated. This modulation in perceived size induced by spatial attention was associated with an increase in activity in the extrastriate region that was greater for size overestimation compared to size underestimation. There was also a strengthening of connection between the extrastriate region and the right precuneus. Further DCM analysis showed that relative to underestimation, overestimation condition significantly increased the extrastriate activity as a consequence of decreased inhibitory self-connection and increased feedback projections from the right precuneus to the extrastriate region.

On a neurophysiological point of view, it is well-known that the density of retinal receptive fields is higher in the fovea than in the periphery and many more neurons are devoted to the processing of central vision (cortical magnification). As a consequence, visual resolution is reduced in the periphery.^18^ Consistent with this, behavioural studies have shown that object size is consistently underestimated in the periphery,^19-22^ suggesting that the number of neurons activated determines perceived size.^23^ The present study provides direct evidence in favour of this hypothesis by showing that the object associated with a larger perceived size also activated a larger region of the early visual cortex in comparison to an identical object associated with a smaller perceived size.

A growing body of evidence indicates that extrastriate and parietal regions are involved in subjective size perception. Several fMRI studies have shown that context-dependent visual size perception is correlated with both anatomical and functional properties of V1.^6,24-27^ Intriguingly, Moutsiana *et al*.^28^ reported that even in the absence of contextual information (and therefore top-down modulation), perceptual biases in size perception correlated with individual differences in the size of the population receptive fields, providing compelling evidence for the role of V1 in size perception. Additional evidence comes from studies using perturbation techniques. It has been reported that activation or disruption of early visual cortex induced by transcranial direct current stimulation and transcranial magnetic stimulation, respectively, can increase or decrease the size illusion effects.^7,29^ Moreover, patients who have lesions involving the striate and extrastriate visual areas fail to exhibit susceptibility to the Müller-Lyer illusion.^30^ In keeping with these findings, our fMRI results confirmed an involvement of the early visual cortex in visual size perception: apparently bigger objects elicited greater activation in the extrastriate region than apparently smaller objects, even if the target had always the same retinal size. Other than early visual areas, the parietal cortex has also been found to be involved in the processing of apparent object size,^31-34^ and both the concentration of inhibitory neurotransmitter (i.e. GABA) and intrinsic activity in the right parietal cortex can predict inter-individual variability in the strength of size illusions.^8,9^

Importantly, occipital and parietal cortices are retinotopically connected to allow for transmission of top-down attention signals from parietal to occipital regions.^35,36^ For instance, it has been reported that activity in extrastriate cortex consistently covaries with responses in the parietal cortex with a right hemispheric dominance during binocular rivalry.^37^ Moreover, the intrinsic forward connectivity from V1 to right parietal cortex negatively predicts the magnitude of the Ebbinghaus illusion.^8^ In line with these findings, our results showed that the connection between early visual cortex and the right parietal region was associated with the processing of the Ebbinghaus illusion. Notably, feedback projection from the parietal region to the early visual cortex was linked to perceived object size, with size overestimation strengthening the connection relative to a physically identical object of smaller perceived size. Taken together, both task-free feedforward connection and task-based feedback connection between the early visual cortex and the right parietal cortex predict apparent object size, but in opposite directions.

Our findings might have important implications for the reduced susceptibility to visual illusions sometimes observed in children with autism spectrum disorder (ASD).^38,39^ Intriguingly, it has been demonstrated that individuals with ASD have decreased functional connectivity between the precuneus and the visual cortex.^40^ Future studies should examine if this connectivity might predict individual differences in the strength of visual illusions.

In summary, the current study unravels the neural mechanisms underlying attentional modulation of apparent object size in the context of the Ebbinghaus illusion. Our findings clearly indicate that the illusion relies on feedback projections from higher- to lower-level visual areas, highlighting the essential role of top-down signals in conscious visual perception. Moreover, we provide further support for the contribution of early visual cortex to context-dependent visual size perception.

## Data Availability

Behavioural and fMRI data used in this study will be made available upon reasonable request to the corresponding authors.

## References

[1] Song C, Schwarzkopf DS, Rees G. Interocular induction of illusory size perception. BMC Neurosci. 2011;12(1):27. 21396093 10.1186/1471-2202-12-27 PMC3062603

[2] Nakashima Y, Sugita Y. Size-contrast illusion induced by unconscious context. J Vis. 2018;18(3):16. 10.1167/18.3.16 29677332

[3] Chen L, Qiao C, Wang Y, Jiang Y. Subconscious processing reveals dissociable contextual modulations of visual size perception. Cognition. 2018;180:259–267. 30096483 10.1016/j.cognition.2018.07.014

[4] Sperandio I, Lak A, Goodale MA. Afterimage size is modulated by size-contrast illusions. J Vis. 2012;12(2):18. 10.1167/12.2.18 22353777

[5] Schwarzkopf DS, Rees G. Subjective size perception depends on central visual cortical magnification in human V1. PLoS One. 2013;8(3):e60550. 23536915 10.1371/journal.pone.0060550 PMC3607553

[6] Schwarzkopf DS, Song C, Rees G. The surface area of human V1 predicts the subjective experience of object size. Nat Neurosci. 2011;14(1):28–30. 21131954 10.1038/nn.2706 PMC3012031

[7] Wang A, Chen L, Jiang Y. Anodal occipital transcranial direct current stimulation enhances perceived visual size illusions. J Cogn Neurosci. 2021;33(3):528–535. 33326330 10.1162/jocn_a_01664

[8] Wu B, Feng B, Han X, Chen L, Luo W. Intrinsic excitability of human right parietal cortex shapes the experienced visual size illusions. Cerebral Cortex. 2023;33(10):6345–6353. 36562991 10.1093/cercor/bhac508

[9] Song C, Sandberg K, Andersen LM, Blicher JU, Rees G. Human occipital and parietal GABA selectively influence visual perception of orientation and size. J Neurosci. 2017;37(37):8929–8937. 28821653 10.1523/JNEUROSCI.3945-16.2017 PMC5597977

[10] Shulman GL . Attentional modulation of size contrast. Q J Exp Psychol. 1992;45(4):529–546. 10.1080/14640749208401332 1484973

[11] Brainard DH . The psychophysics toolbox. Spat Vision. 1997;10:433–436. 9176952

[12] Pelli DG . The VideoToolbox software for visual psychophysics: Transforming numbers into movies. Spat Vision. 1997;10(4):437–442. 9176953

[13] Weidner R, Boers F, Mathiak K, Dammers J, Fink GR. The temporal dynamics of the Müller-Lyer illusion. Cerebral Cortex. 2010;20(7):1586–1595. 19875676 10.1093/cercor/bhp217

[14] Friston K, Buechel C, Fink G, Morris J, Rolls E, Dolan R. Psychophysiological and modulatory interactions in neuroimaging. Neuroimage. 1997;6(3):218–229. 9344826 10.1006/nimg.1997.0291

[15] Lumaca M, Dietz MJ, Hansen NC, Quiroga-Martinez DR, Vuust P. Perceptual learning of tone patterns changes the effective connectivity between Heschl’s gyrus and planum temporale. Hum Brain Mapp. 2021;42(4):941–952. 33146455 10.1002/hbm.25269 PMC7856650

[16] Cunningham SI, Tomasi D, Volkow ND. Structural and functional connectivity of the precuneus and thalamus to the default mode network. Hum Brain Mapp. 2017;38(2):938–956. 27739612 10.1002/hbm.23429 PMC6866740

[17] Cavanna AE, Trimble MR. The precuneus: A review of its functional anatomy and behavioural correlates. Brain. 2006;129(Pt 3):564–583. 16399806 10.1093/brain/awl004

[18] Cowey A, Rolls ET. Human cortical magnification factor and its relation to visual acuity. Exp Brain Res. 1974;21(5):447–454. 4442497 10.1007/BF00237163

[19] Newsome LR . Visual angle and apparent size of objects in peripheral vision. Percept Psychophys. 1972;12(3):300–304.

[20] Anstis S . Picturing peripheral acuity. Perception. 1998;27(7):817–825. 10209644 10.1068/p270817

[21] Baldwin J, Burleigh A, Pepperell R, Ruta N. The perceived size and shape of objects in peripheral vision. Iperception. 2016;7(4):2041669516661900. 10.1177/2041669516661900 PMC5030758 27698981

[22] Kirsch W, Pfister R, Kunde W. On why objects appear smaller in the visual periphery. Psychol Sci. 2020;31(1):88–96. 31829781 10.1177/0956797619892624

[23] Han Y, Tan Z, Zhuang H, Qian J. Contrasting effects of exogenous and endogenous attention on size perception. Br J Psychol. 2022;113(1):153–175. 34435351 10.1111/bjop.12529

[24] Fang F, Boyaci H, Kersten D, Murray SO. Attention-dependent representation of a size illusion in human V1. Curr Biol. 2008;18(21):1707–1712. 18993076 10.1016/j.cub.2008.09.025 PMC2638992

[25] Sperandio I, Chouinard PA, Goodale MA. Retinotopic activity in V1 reflects the perceived and not the retinal size of an afterimage. Nat Neurosci. 2012;15(4):540–542. 22406550 10.1038/nn.3069

[26] Murray SO, Boyaci H, Kersten D. The representation of perceived angular size in human primary visual cortex. Nat Neurosci. 2006;9(3):429–434. 16462737 10.1038/nn1641

[27] Pooresmaeili A, Arrighi R, Biagi L, Morrone MC. Blood oxygen level-dependent activation of the primary visual cortex predicts size adaptation illusion. J Neurosci. 2013;33(40):15999–16008. 24089504 10.1523/JNEUROSCI.1770-13.2013 PMC4888977

[28] Moutsiana C, de Haas B, Papageorgiou A, et al Cortical idiosyncrasies predict the perception of object size. Nat Commun. 2016;7:12110. 27357864 10.1038/ncomms12110 PMC4931347

[29] Zeng H, Fink GR, Weidner R. Visual size processing in early visual cortex follows lateral occipital cortex involvement. J Neurosci. 2020;40(22):4410–4417. 32350038 10.1523/JNEUROSCI.2437-19.2020 PMC7252478

[30] Daini R, Angelelli P, Antonucci G, Cappa SF, Vallar G. Exploring the syndrome of spatial unilateral neglect through an illusion of length. Exp Brain Res. 2002;144(2):224–237. 12012160 10.1007/s00221-002-1034-8

[31] Plewan T, Weidner R, Eickhoff SB, Fink GR. Ventral and dorsal stream interactions during the perception of the Müller-Lyer illusion: Evidence derived from fMRI and dynamic causal modeling. J Cogn Neurosci. 2012;24(10):2015–2029. 22721374 10.1162/jocn_a_00258

[32] Harvey BM, Fracasso A, Petridou N, Dumoulin SO. Topographic representations of object size and relationships with numerosity reveal generalized quantity processing in human parietal cortex. Proc Natl Acad Sci. 2015;112(44):13525–13530. 26483452 10.1073/pnas.1515414112 PMC4640722

[33] Borghesani V, de Hevia MD, Viarouge A, Pinheiro-Chagas P, Eger E, Piazza M. Processing number and length in the parietal cortex: Sharing resources, not a common code. Cortex. 2019;114:17–27. 30219571 10.1016/j.cortex.2018.07.017

[34] Weidner R, Fink GR. The neural mechanisms underlying the Müller-Lyer illusion and its interaction with visuospatial judgments. Cerebral Cortex. 2007;17(4):878–884. 16707733 10.1093/cercor/bhk042

[35] Silver MA, Kastner S. Topographic maps in human frontal and parietal cortex. Trends Cogn Sci (Regul Ed). 2009;13(11):488–495. 10.1016/j.tics.2009.08.005 PMC2767426 19758835

[36] Romei V, Gross J, Thut G. On the role of prestimulus alpha rhythms over occipito-parietal areas in visual input regulation: Correlation or causation? J Neurosci. 2010;30(25):8692–8697. 20573914 10.1523/JNEUROSCI.0160-10.2010 PMC6634639

[37] Lumer ED, Rees G. Covariation of activity in visual and prefrontal cortex associated with subjective visual perception. Proc Natl Acad Sci. 1999;96(4):1669–1673. 9990082 10.1073/pnas.96.4.1669 PMC15554

[38] Happé FG . Studying weak central coherence at low levels: Children with autism do not succumb to visual illusions. A research note. J Child Psychol Psychiatry. 1996;37(7):873–877. 8923230 10.1111/j.1469-7610.1996.tb01483.x

[39] Bölte S, Holtmann M, Poustka F, Scheurich A, Schmidt L. Gestalt perception and local-global processing in high-functioning autism. J Autism Dev Disord. 2007;37(8):1493–1504. 17029017 10.1007/s10803-006-0231-x

[40] Lynch CJ, Uddin LQ, Supekar K, Khouzam A, Phillips J, Menon V. Default mode network in childhood autism: Posteromedial cortex heterogeneity and relationship with social deficits. Biol Psychiatry. 2013;74(3):212–219. 23375976 10.1016/j.biopsych.2012.12.013 PMC3710546

